# Specialised emission pattern of leaf trace in a late Permian (253 million-years old) conifer

**DOI:** 10.1038/srep12405

**Published:** 2015-07-22

**Authors:** Hai-Bo Wei, Zhuo Feng, Ji-Yuan Yang, Yu-Xuan Chen, Jia-Jia Shen, Xiao-Yuan He

**Affiliations:** 1 Yunnan Key Laboratory for Palaeobiology, Yunnan University, Kunming 650091, China; 2 State Key Laboratory of Palaeobiology and Stratigraphy, Nanjing Institute of Geology and Palaeontology, Chinese Academy of Sciences, Nanjing 210008, China

## Abstract

Leaf traces are important structures in higher plants that connect leaves and the stem vascular system. The anatomy and emission pattern of leaf traces are well studied in extant vascular plants, but remain poorly understood in fossil lineages. We quantitatively analysed the leaf traces in the late Permian conifer *Ningxiaites specialis* from Northwest China based on serial sections through pith, primary and secondary xylems. A complete leaf traces emission pattern of a conifer is presented for the first time from the late Palaeozoic. Three to five monarch leaf traces are grouped in clusters, arranged in a helical phyllotaxis. The leaf traces in each cluster can be divided into upper, middle and lower portions, and initiate at the pith periphery and cross the wood horizontally. The upper leaf trace increases its diameter during the first growth increment and then diminishes completely, which indicates leaf abscission at the end of the first year. The middle trace immediately bifurcates once or twice to form two or three vascular bundles. The lower trace persists as a single bundle during its entire length. The intricate leaf trace dynamics indicates this fossil plant had a novel evolutionary habit by promoting photosynthetic capability for the matured plant.

Leaves are important functional organs in most vascular plants for nutrients supply by mean of photosynthesis^1^. They attached at the node of the plant axis, where one or more vascular bundles diverge into the base of each leaf. These divergent bundles are termed “leaf traces”, by contrast to the so-called sympodial bundles, which continue their course through the next internode^2^,^3^. A leaf trace is a thin vascular bundle in the stem, extending between its connection with a leaf and that with another vascular unit in the stem^4^. The emission of leaf vasculature and developmental pattern in extant gymnosperms have been largely described and are well understood, however, relatively few detailed analyses of leaf traces exist for their fossil lineages^5^,^6^,^7^.

Comparative investigations on fossil plants of various ages have contributed significantly to the understanding of the development and evolution of the primary vascular systems of seed plants^8^,^9^,^10^,^11^. Previous study of leaf traces in Palaeozoic arborescent seed plants mainly relate to manoxylic medullosan pteridosperms^12^,^13^,^14^,^15^,^16^,^17^,^18^ and pycnoxylic cordaitaleans gymnosperms^19^,^20^,^21^,^22^,^23^,^24^,^25^. In contrast, Palaeozoic conifers and conifer-like plants are scarcely discussed^26^,^27^, which may be due to the paucity of appropriate material.

Here, we present a detailed emission pattern of leaf traces in the late Permian conifer *Ningxiaites specialis* Feng from Northwest China by using quantitative analyses of leaf traces on serial tangential thin sections. The leaf traces originate at the margin of the pith and bifurcate once or twice, or persist as a single vascular bundle extension. They are grouped in regular clusters arranged in a helical phyllotaxis on the plant stem. Each leaf trace cluster consists of three to five leaf traces. One of the leaf traces in the cluster disappears at the end of the first year’s growth, while other traces in the cluster persist and penetrate horizontally through the woody cylinder with slightly increased diameter. Our study provides a better understanding of the physiological feature (leaf phenology, leaf trace dynamics) of a primitive Palaeozoic conifer.

## Results

**Figure 1:**
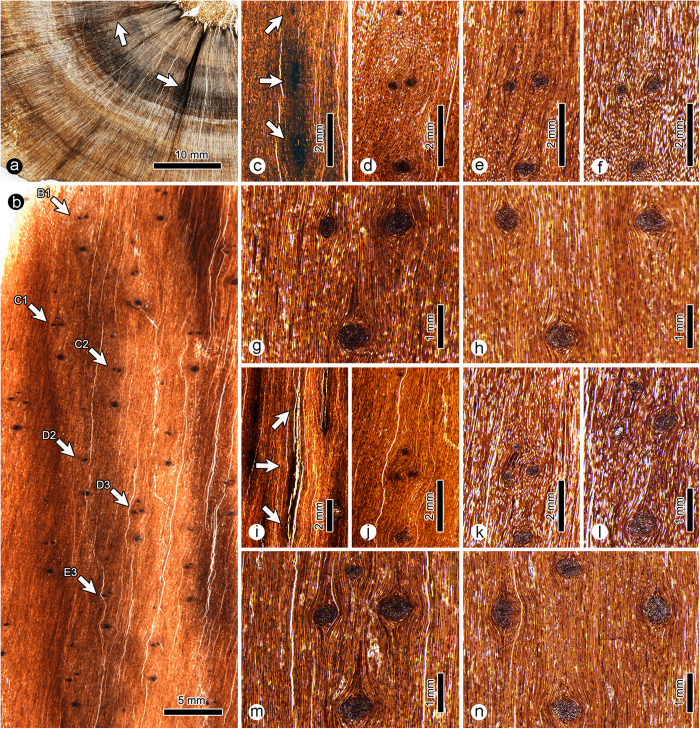
*Ningxiaites specialis* Feng from the upper Permian of Northwest China. (**a**), Transverse section showing the bifurcation of the leaf trace and the horizontal extension of leaf traces (arrows). (**b**), Tangential section showing the helical arrangement of leaf trace clusters; B1, C1, C2, D2, D3 and E3 represent leaf trace clusters. **(c–h**), showing the leaf trace cluster B1 in different thin sections, arrows indicate leaf traces. (**i–j**), showing the leaf trace cluster C5 in different thin sections, arrows indicate leaf traces.

The silicious permineralised plant of this study was obtained from the late Permian (ca 253 million-years old, Changhsingian) Sunjiagou Formation in Shitanjing Coalfield, Ningxia Huizu Autonomous Region, China. The plant axis has been fragmented by weathering into blocks of various dimensions. Taxonomically, it was assigned to *Ningxiaites specialis* Feng with a putative coniferous affinity^26^. The species is characterised by parenchymatous pith, endarch primary xylem and pycnoxylic secondary xylem. The species is especially characterised by the occurrence of pits on the end (tangential) walls of ray cells and the inflated cells in the secondary xylem. Leaf traces initiate at the margin of pith and are separated by numerous xylem strands (Fig. 1a, arrows).

**Figure 2:**
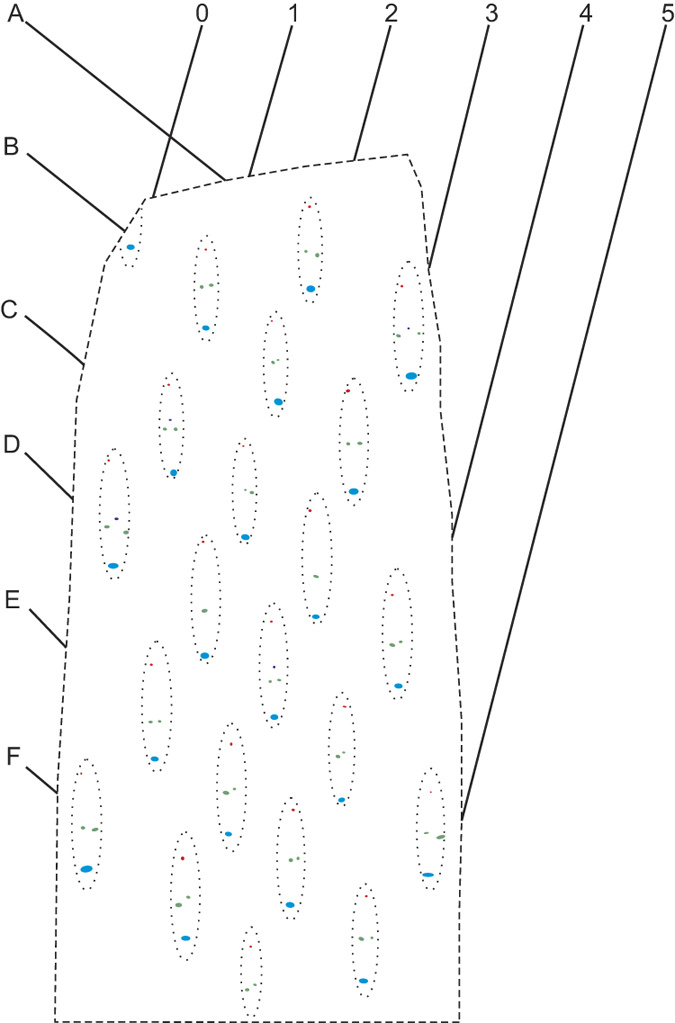
Coordination of the helically arranged leaf trace clusters in *Ningxiaites specialis* Feng.

**Figure 3:**
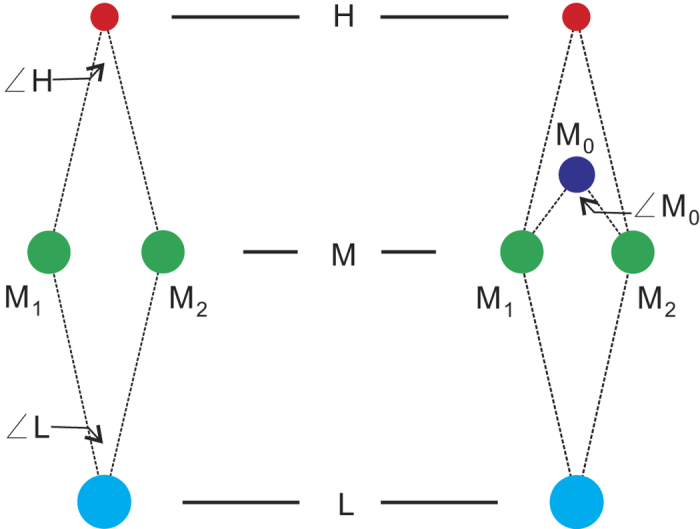
Leaf traces in the spindle leaf trace cluster in *Ningxiaites specialis* Feng. H, M and L represent the upper, middle and lower portions in the leaf trace cluster; M_0_, M_1_ and M_2_ represent the divided trace bundles of M. ∠H, ∠M_0_ and ∠L represent the angles of M_1_HM_2_, M_1_M_0_M_2_ and M_1_LM_2_.

The leaves are helically arranged on the plant axis based on the arrangement of leaves traces. Examination of thin sections reveals that each leaf trace of *Ningxiaites specialis* has only one monarch vascular bundle. Three to five leaf traces are grouped in a cluster, which is of vertically elongated elliptical shape and helically arranged on the stem (Fig. 1b, arrows; Fig. 2). The leaf traces in a cluster can be divided into upper (H), middle (M) and lower (L) portions (Fig. 3). There is only one leaf trace in the upper and lower portions respectively. The middle portion consists of one leaf trace initially, at the pith margin, it then bifurcates once or twice forming two or three leaf traces (M_0_, M_1_ and M_2_) (Fig. 3).

**Figure 4:**
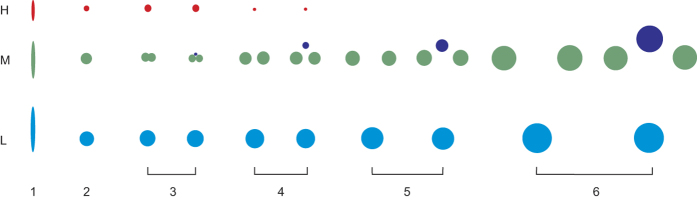
The emission stages of the leaf trace in *Ningxiaites specialis* Feng. H, M and L represent the upper, middle and lower portions in the leaf trace cluster. Stage 1 represents the initials of the leaf traces. Stage 2 represents the undivided leaf traces. Stage 3 represents the end of the first year’s growth. Stages 4 to 6 show that the leaf traces slightly increase their diameter during their entire extent course.

The eighteen serial tangential thin sections have been examined for the emission pattern of the leaf traces. Thin section No. I shows the structure of the pith, which consists of vertical files of parenchymatous cells. Thin section II represents the transitional region between pith and xylem: leaf trace initials (H, M and L) arranged vertically in a cluster can be recognised in this stage (Fig. 4). The diameter of each leaf trace in this stage is very small, although they appear to be superficially larger and of vertically elongate form. Thin sections III–XVIII show nearly mature leaf traces in each cluster. Thus, if H and M_0_ are present, each leaf trace cluster has five leaf traces, and if H and M_0_ are absent, each cluster has three leaf traces (Fig. 4).

**Figure 5:**
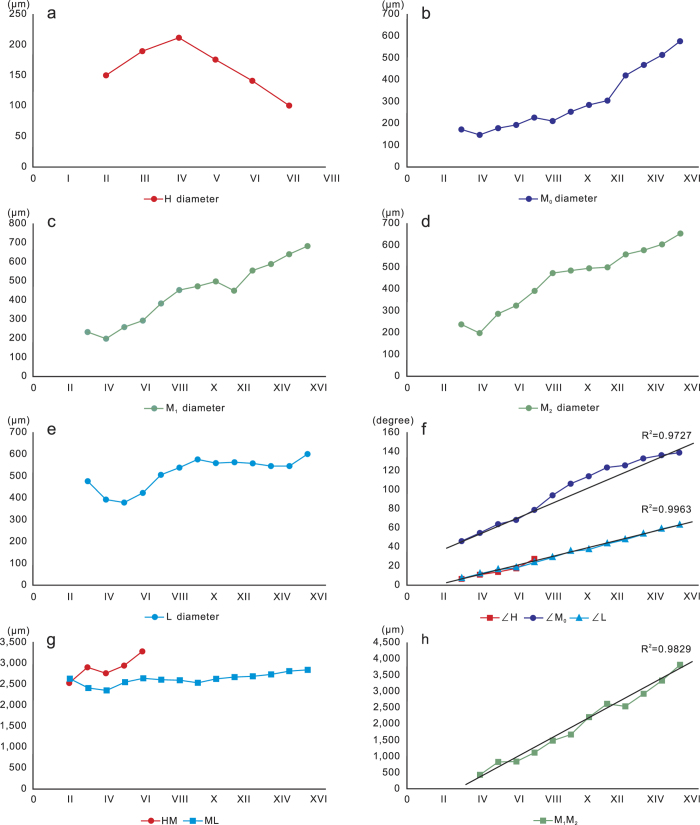
Statistics of leaf traces in *Ningxiaites specialis* Feng. Numbers (I–XVIII) of horizontal coordinates represent the numbers of the thin sections.

Leaf trace H is located in an uppermost position in the leaf trace cluster. It is present only in the first two growth rings. From an initial diameter of less than 100 μm H reaches a maximal diameter of nearly 300 μm in thin section IV. In thin sections V and VI, the diameter of trace H gradually becomes narrower (Fig. 5a), and then completely disappeared. The last occurrence of H is seen in B1 of thin section VII.

Leaf trace M is located at a middle position in the leaf trace cluster. It immediately bifurcates once or twice after emerging from the pith margin. The initial diameter of M is ca 190 μm. Where it bifurcates once, the trace divides into two horizontally arranged bundles (M_1_ and M_2_) having nearly equal diameters (Fig. 1c–h). Where it bifurcates twice, trace M firstly divides into two vertically arranged bundles, the upper one (M_0_) having a much smaller diameter than the lower bundle. The relatively large-diameter lower bundle then bifurcates into two horizontally arranged bundles (M_1_ and M_2_) having nearly equal diameters (Fig. 1i–n). Traces M_0_ (if present), M_1_ and M_2_ cross the wood cylinder horizontally with gradually increased diameters (Fig. 5b–d).

Leaf trace L is located at the lowermost position in the leaf trace cluster. It persists as a single vascular bundle and crosses the wood cylinder horizontally. The initial diameter of trace L is apparently larger than that of H and M, and gradually increases from ca 250 μm to 750 μm (Fig. 5e).

The diameter of leaf traces M_0_, M_1_, M_2_ and L are at their minimal in thin section IV (Fig. 5b–e), and gradually increase in diameter in onward sections.

The angles of ∠H, ∠M_0_ and ∠L increase according to linear relationships (R^2^ = 0.973–0.996) during growth (Fig. 5f).

The vertical distances between traces H and M, and M and L through the thin sections show little difference (Fig. 5g). The horizontal distance between traces M_1_ and M_2_ changes in a linear relationship (R^2^ = 0.983) (Fig. 5h).

## Discussion

Fossil plants with preserved anatomy such as the specimen described herein represent an exceptional source of information on organ development in extinct plant taxa. We present the first detailed leaf trace emission pattern, in a late Permian conifer from Northwest China. Based on serial thin sections of leaf traces we document for the first time two additional distinctive traits of *Ningxiaites specialis*: the unique bifurcation pattern of leaf traces, and that leaves have different life spans.

Based on a comparative investigation of the primary vascular systems of living conifers, Namboodiri and Beck^5^,^6^,^7^ concluded that in those species with helical phyllotaxis the leaf trace supplying a leaf is single at the point of origin, whereas in species with opposite leaves two traces originate from two individual bundles and fuse to form the single strand supplying a leaf. Our specimen shows helically arranged leaf traces. This implies that each trace should represent a single strand supplying a leaf. Because of the arrangement and the small diameter of these traces, the possibility of dormant buds, protective bracts or epicormic shoot/branch traces can be excluded.

It is worth mentioning that the upper leaf trace in our specimen persists into the second growth ring, however, due to the apparent change of its diameter the portion in the second year’s increment may belong to the remnant of leaf trace in bark while leaf abscission. Whereas, the middle and lower leaf traces penetrating more than ten growth rings are observed. Thus, two forms of leaf longevity were speculated in *Ningxiaites specialis*, from a one-year to many years life span. Some leaves were partially shed off at the end of the first year’s growth, but the majority of leaves persisted over many years. Thus, the plant had an evergreen canopy although the leaf morphology of the plant is unknown at present. However, this foliar habit may have been beneficial in leaving more space between each leaf cluster and even for leaves within a single cluster due to the fact that a “one-year” leaf overlaps with other leaves in the clusters. Therefore, the rest of leaves had a better photosynthetic, nutrient gaining capacity during growth.

The statistical analyses show that the middle and lower leaf traces in the leaf trace clusters increased their diameters when the upper leaf trace fell at the end of the first year’s growth. This phenomenon indicates that the plant received more photosynthetic products from the upper leaf than other leaves during the first year, but after the abscission of the upper leaf, the middle and lower leaves in the leaf clusters supplied increased products to the plant.

Amongst Palaeozoic pycnoxylic gymnosperms in both Euramerican and Cathaysian floras the phenomenon of bifurcation of leaf trace is better understood for cordaitales, especially based on coal ball materials^20^,^28^,^29^. However, the modes of leaf trace division of those plants are quite distinct from that of the plant described herein.

In the Carboniferous *Cordaixylon birame* and *C. iowense* (sensu Trivett^20^) from North America, leaf traces are always single from the margin of the pith to the end of xylem cylinder, but they do divide in the cortex to form two bundles^20^. In *C. dumusum* the leaf trace is single at the margin of the pith in smaller stems, but it is double in many larger stems^29^. Whereas in species of *Mesoxylon*, leaf traces also originate at the pith margin but immediately divide into two bundles at the pith margin^28^.

In the early Permian *Shanxioxylon sinense* from North China leaf traces diverge from the pith margin as single bundles, then extend nearly horizontally through the secondary xylem. They bend abruptly upward where leaf traces pass through the phloem and cortex, and divide once into double strands in the outer cortex^21^,^23^. The leaf traces of *Cordaixylon tianii* either frequently divide immediately into two bundles at the margin of the pith or occasionally remain as a single trace after they have diverged from the pith^21^,^24^. However, in *Shanxioxylon taiyuanense* the leaf traces diverge from the pith margin as double (less frequently singly) bundles^21^,^25^.

Leaf trace emission patterns of Chinese Palaeozoic conifers and conifer-like plants rarely have been studied. Previously documented species have their leaf traces rising from the periphery of the pith as single vascular bundles, then extending outwards nearly horizontally through the secondary xylem, and arranged helically on the plant stem.

Both the middle Permian (Roadian–Wordian) *Plyophyllioxylon hulstaiense*^30^ and the late Permian (Changhsingian) *Shenoxylon mirabile*^31^ from the same region as the specimen studied herein, possess dense helically arranged leaf traces which also cross the xylem cylinder horizontally. However, bifurcation of leaf traces is not recognised from those two conifer-like species.

*Szecladia multinervia* was reported from the upper Permian (Wuchiapingian) of South China of putative podocarpaceous affinity^32^. Leaf traces in this plant differ from our species in diverging from the stele as a single bundle, and then dividing several times in the cortex and at the base of the leaves, forming seven or eight parallel veins in each leaf.

He *et al*.^27^ described a very distinctive plant, *Xuanweioxylon scalariforme* from the upper Permian (Wuchiapingian) of Southwest China. Apart from the scalariform thickenings of tracheids in the secondary xylem, the primary vascular system of this enigmatic plant consists of only leaf traces. Leaf traces originate at the pith periphery as single strands and commonly divide to form double traces and extend through the secondary xylem. Sometimes some leaf traces may divide to form 3–4 vascular bundles. Like other Palaeozoic conifer and conifer-like plants, leaf traces in *Xuanweioxylon* are helically arranged, but all of the leaf traces are occluded after a few millimeters, which indicates a deciduous habit.

Other conifer and conifer-like plants, for example *Guizhouoxylon dahebianense*^33^ and *Walchiopremnon gaoi*^34^, have been reported from the late Permian (Wuchiapingian) deposits of Southwest China. However, because their anatomical structure is poorly preserved, detailed comparison of leaf trace emission with *Ningxiaites specialis* is not possible.

Deciduous characteristic is an innovation in Palaeozoic seed plant evolution. Evidences for a deciduous habit have been provided from Carboniferous-Permian progymnopserm including *Archaeopteris*^35^, arborescent pycnoxylic pteridosperms^36^, cordaitaleans including *Mesoxylon thompsonii*^19^ and *Mesoxylon* sp^37^ and presumably coniferophytes such as *Xuanweioxylon*^27^. In contrast to shedding off single leaf, some Palaeozoic Walchiaceae conifers have been proved that they shed off leafy twigs or even complete branching systems like modern aracarians do^38^,^39^. One leaf trace (H) in the leaf trace cluster of *Ningxiaites specialis* disappeared during its extension, indicating that this plant is partially deciduous. *Ningxiaites specialis* could represent a novel evolutionary form that has an evergreen canopy but sheds off some leaves from regular positions at one-year old.

## Conclusions

The first detailed leaf trace emission pattern is described in a late Permian conifer plant, based on a study of *Ningxiaites specialis* Feng from Northwest China. Quantitative analyses of leaf traces through serial sections reveal that three to five monarch leaf traces are grouped in clusters arranged in a helical pattern. The leaf traces in each cluster can be divided into three portions, all of which initiate at the pith periphery and cross the wood horizontally. The upper leaf trace abscised at the end of the first year. The middle trace immediately bifurcates once or twice to form two or three vascular bundles. The lower trace persists as a single bundle throughout its entire extent. The complex leaf trace dynamics shows that *N. specialis* had a distinctive evolutionary habit that facilitated increased photosynthesis capability in the matured plant.

## Methods

In order to examine the emission pattern of the leaf traces, one well-preserved wedge-shaped specimen, 60 mm high × 50 mm wide, including pith, primary and secondary xylem was sectioned by serial thin section technique. Thin sections along transverse and tangential planes were prepared as follows. The specimen was sectioned to appropriately thin wafers using a diamond saw and the upper surfaces were ground using a grinding wheel with carborundum grit in a decreasing series of #240, #400 and #800 grade sizes. The smooth upper surfaces were then attached to glass slides with Buehler EpoThin^TM^ Epoxy Resin (20-8140-032) and EpoThin^TM^ Epoxy Hardener (20-8142-016), and the exposed surfaces were ground to a thickness of 30–50 μm. A total of eighteen tangential thin sections (Nos. I–XVIII) were made through the pith, primary xylem and secondary xylem with a thickness of ca 2.5 mm between each thin section.

Leaf trace clusters in the thin sections are coordinated as A to F from top to bottom and 0 to 5 from right to left, and named as follows: A1, A2, A3, B0, B1, B2, B3, B4, B5, C0, C1 ,C2, C3 ,C4, D1, D2, D3, D4, D5, E1, E2, E3, E4, F1, F2, F3, F4 and F5 (Fig. 2). Thus, each leaf trace cluster has a unique coordinate number to trace through all thin sections. Because there are three to five leaf traces present in each leaf trace cluster, we denoted leaf traces in a different potion of a cluster as H, M (including M_0_, M_1_ and M_2_) and L (Fig. 3).

More than two thousands measurements, including the diameter of each leaf trace, angles and the distances between each leaf trace in a cluster, have been obtained from the eighteen thin sections. Amongst the total of twenty-nine leaf trace clusters that are maximally assessable on the thin sections, six typical leaf trace clusters (B1, C1, C2, D2, D3, E3) located in the central region on the thin sections and traceable through all eighteen thin sections, were used for the quantitative analyses of the emission pattern of the leaf traces. Of these, only B1 and D3 of different forms of leaf trace emission patterns are illustrated in the current paper.

Measurements were made directly on digital images by Leica Application Suite ver. 4.2.0 program to an accuracy of 1 μm. Final plots with mean data were utilised by applying statistic software SPSS.

Optical examination and photomicrographs were undertaken by using a Leica DM 2500 M transmitted light microscope and a Leica M205 C stereoscopic microscope, both equipped with a Leica DFC 500 digital camera. Images in plates were stitched together using Adobe Photoshop CS 5 Extended, and the line drawing was made in CorelDRAW Graphics Suite X4. Specimen and thin sections are housed in the Palaeobotanical Collections of the Yunnan Key Laboratory for Palaeobiology, Yunnan University, China, and have the catalogue number YKLP20010.

## Additional Information

**How to cite this article**: Wei, H.-B. *et al*. Specialised emission pattern of leaf trace in a late Permian (253 million-years old) conifer. *Sci. Rep*. **5**, 12405; doi: 10.1038/srep12405 (2015).
